# Reductions in Corpus Callosum Volume Partially Mediate Effects of Prenatal Alcohol Exposure on IQ

**DOI:** 10.3389/fnana.2017.00132

**Published:** 2018-01-12

**Authors:** Stevie C. Biffen, Christopher M. R. Warton, Nadine M. Lindinger, Steven R. Randall, Catherine E. Lewis, Christopher D. Molteno, Joseph L. Jacobson, Sandra W. Jacobson, Ernesta M. Meintjes

**Affiliations:** ^1^Department of Human Biology, Faculty of Health Sciences, University of Cape Town, Cape Town, South Africa; ^2^Department of Psychiatry and Mental Health, Faculty of Health Sciences, University of Cape Town, Cape Town, South Africa; ^3^Department of Psychiatry and Behavioral Neurosciences, Wayne State University School of Medicine, Detroit, MI, United States; ^4^MRC/UCT Medical Imaging Research Unit, Division of Biomedical Engineering, Department of Human Biology, Faculty of Health Sciences, University of Cape Town, Cape Town, South Africa

**Keywords:** fetal alcohol spectrum disorders, MRI, subcortical volumes, corpus callosum, IQ

## Abstract

Disproportionate volume reductions in the basal ganglia, corpus callosum (CC) and hippocampus have been reported in children with prenatal alcohol exposure (PAE). However, few studies have investigated these reductions in high prevalence communities, such as the Western Cape Province of South Africa, and only one study made use of manual tracing, the gold standard of volumetric analysis. The present study examined the effects of PAE on subcortical neuroanatomy using manual tracing and the relation of volumetric reductions in these regions to IQ and performance on the California Verbal Learning Test-Children's Version (CVLT-C), a list learning task sensitive to PAE. High-resolution T1-weighted images were acquired, using a sequence optimized for morphometric neuroanatomical analysis, on a Siemens 3T Allegra MRI scanner from 71 right-handed, 9- to 11-year-old children [9 fetal alcohol syndrome (FAS), 19 partial FAS (PFAS), 24 non-syndromal heavily exposed (HE) and 19 non-exposed controls]. Frequency of maternal drinking was ascertained prospectively during pregnancy using timeline follow-back interviews. PAE was examined in relation to volumes of the CC and left and right caudate nuclei, nucleus accumbens and hippocampi. All structures were manually traced using Multitracer. Higher levels of PAE were associated with reductions in CC volume after adjustment for TIV. Although the effect of PAE on CC was confounded with smoking and lead exposure, additional analyses showed that it was not accounted for by these exposures. Amongst dysmorphic children, smaller CC was associated with poorer IQ and CVLT-C scores and statistically mediated the effect of PAE on IQ. In addition, higher levels of PAE were associated with bilateral volume reductions in caudate nuclei and hippocampi, effects that remained significant after control for TIV, child sex and age, socioeconomic status, maternal smoking during pregnancy, and childhood lead exposure. These data confirm previous findings showing that PAE is associated with decreases in subcortical volumes and is the first study to show that decreases in callosal volume may play a role in fetal alcohol-related impairment in cognitive function seen in childhood.

## Introduction

Prenatal alcohol exposure (PAE) is associated with a range of neurocognitive and behavioral problems. Fetal alcohol spectrum disorders (FASD) is an umbrella term under which the entire spectrum of outcomes related to PAE falls. Fetal alcohol syndrome (FAS), the most severe of the FASD, is characterized by growth deficits, small head circumference, distinctive craniofacial dysmorphology (small palpebral fissures, flattened philtrum, and thin vermillion) and cognitive problems that relate to specific brain abnormalities (Jones and Smith, 1973; Hoyme et al., 2005). Partial FAS (PFAS) is diagnosed in individuals exhibiting the facial dysmorphology characteristic of FAS, whose mothers are known to have drunk heavily during pregnancy and who exhibit growth deficits, small head circumference or neurobehavioral impairment (Hoyme et al., 2005). Adverse effects on brain development range from microstructural, neurochemical and cellular dysfunction (such as widespread apoptotic neurodegeneration that can result in the death of millions of cells in the forebrain during gestation) to gross structural abnormalities (Ikonomidou, 2000; Olney, 2004; Spadoni et al., 2007; Lebel et al., 2008).

In the U.S. and in Western countries, FAS occurs at a rate estimated to be between 0.05 and 7 per 1,000 live births (May and Gossage, 2001; May et al., 2009). In contrast, certain high-risk communities in South Africa have been found to have an FAS prevalence rating that is 18–148 times greater than in the U.S. (May et al., 2000; Viljoen et al., 2005; Urban et al., 2008). Within the Western Cape province of South Africa, rates are highest amongst the Colored population of mixed Asian, African and European ancestry (May et al., 2000, 2005, 2013; Viljoen et al., 2005).

Magnetic resonance imaging (MRI) allows for non-invasive and quantitative investigation of the structural and functional brain alterations that are observed in FASD. Brain regions that have been consistently implicated in FASD include the corpus callosum (CC), cerebellum, parietal lobe, frontal lobe, temporal lobe, caudate nucleus, hippocampus and increased volume of lateral ventricles (see review papers, Lebel et al., 2011; Donald et al., 2015). While most regions show alcohol-related volume reductions, findings in certain subcortical brain regions have not always been consistent. For example, some studies report bilateral decreases in hippocampal volume (Willoughby et al., 2008; Astley et al., 2009; Coles et al., 2011; Nardelli et al., 2011; Treit et al., 2013) with PAE, while others find relative sparing (Archibald et al., 2001), or even volume increases (Riikonen et al., 2007). Methodological differences, such as automated vs. manual segmentation, failure or different strategies to control for total intracranial volume (TIV), small sample sizes, or large age ranges may explain some of these differences. Notably, one study found increased hippocampal volume relative to brain volume with age (9–15 years) in controls, but not in children with FASD (Willoughby et al., 2008), highlighting the potential contribution of age-related changes in brain development when comparing diagnostic groups and the need for narrow age ranges. It is not yet known to what extent developmental trajectories in children prenatally exposed to alcohol are altered.

Although manual tracing is considered the gold standard for volumetric analysis, yielding the greatest consistency and sensitivity (Morey et al., 2009), few studies employ this method due to high time demands (Archibald et al., 2001; Riikonen et al., 2007; Willoughby et al., 2008; Astley et al., 2009). Here we use manual tracing of study participants within a narrow age range (10.4 ± 0.4 years) to examine the effects of PAE on volumes of select subcortical structures and the CC in 81 children from the Cape Town Longitudinal Cohort (Jacobson et al., 2008), for whom diagnostic and detailed prospective prenatal alcohol and drug exposure data are available. Most previous studies have only been able to examine volumetric differences between diagnostic groups and not dose-dependent effects, since the amount of alcohol used during pregnancy is difficult to recall reliably in retrospective case-controlled studies (Jacobson et al., 2002).

Due to the labor intensive nature of manual tracing, only subcortical structures previously implicated in FASD were investigated (Archibald et al., 2001; Morey et al., 2009; Nardelli et al., 2011). These include the caudate nucleus and hippocampus, which are involved in cognition, memory and emotional networks (Devinsky and D'Esposito, 2004) that are amongst the most affected domains in FASD (Uecker and Nadel, 1996; Mattson et al., 1998; Willoughby et al., 2008; Green et al., 2009; Jacobson et al., 2011; Lewis et al., 2016). Although findings in the nucleus accumbens have rarely been reported in FASD (Archibald et al., 2001), we report it here because the manual tracing protocol we used for the caudate initially involved tracing both structures together. The CC was included as it is clearly visible on MRIs, is easy to delineate, and has been consistently shown to be reduced in FASD (Mattson et al., 1992; Sowell et al., 2001, 2008; Bookstein et al., 2002; Autti-Rämö et al., 2007; Lebel et al., 2008; Astley et al., 2009), even in newborns (Jacobson et al., 2017). PAE has been linked to a larger angle of the splenium (Bookstein et al., 2007), reductions in CC thickness and area in the splenium and anterior third (Yang et al., 2012), and CC volume reductions in the splenium, genu and area just anterior to the splenium (Riley et al., 1995).

In addition, we examined whether alcohol-related regional volumetric changes mediate the effect of PAE on cognitive performance on the Wechsler Intelligence Scale for Children-Fourth Edition (WISC-IV) and the California Verbal Learning Test-Children's Version (CVLT-C). Poorer IQ is frequently seen in FASD (Streissguth et al., 1990, 1991; Jacobson et al., 2004) as is impaired performance on the CVLT-C (Delis et al., 1994), a list learning task (Kerns et al., 1997; Mattson et al., 1998; Crocker et al., 2011; Vaurio et al., 2011; Suttie et al., 2013; Lewis et al., 2015).

We hypothesized that increasing PAE would be associated with volumetric reductions in all regions examined and that these effects would remain significant after controlling for reductions in total brain volume. We also hypothesized that volumetric reductions in hippocampus, caudate nucleus and CC would be associated with poorer WISC IQ and CVLT-C performance and that the effects of PAE on these outcomes would be partially mediated by volumetric changes in the regions that were traced.

## Methods

### Participants

From 1998 to 2002 pregnant women were recruited into the Cape Town Longitudinal Cohort (Jacobson et al., 2008) from an antenatal clinic in a Cape Colored community in Cape Town known to have a high prevalence of alcohol consumption (Croxford and Viljoen, 1999). Detailed alcohol exposure data collected prospectively during pregnancy were available for all of these children. Mothers were interviewed at enrollment and at two subsequent visits using the timeline follow-back (TLFB) approach to record alcohol consumption during pregnancy (Jacobson et al., 2002). We adapted the interview to include information about the type of beverage consumed, container size (including pictures of different containers, bottles, cans, glass size) and sharing (size of container divided by number of women drinking together) to reflect how pregnant women in this community tend to drink. The mother was asked about her drinking on a day-by-day basis during a typical 2-week period around the time of conception, with recall linked to specific times of day and activities. If her drinking had changed since conception, she was also asked about her drinking during the past 2 weeks and when her drinking had changed. At the two follow-up visits, the mother was again asked about her drinking during the previous 2 weeks. Volume was recorded for each type of alcohol beverage consumed each day and converted to oz of absolute alcohol (AA) using multipliers proposed by Bowman et al. (1975) (liquor—0.4, beer—0.05, wine—0.12, cider—0.06). Six summary measures were constructed—average oz AA per day at conception, AA per day averaged across pregnancy, AA per drinking day (quantity per occasion) at conception and across pregnancy, and number of drinking days per week (frequency) at conception and across pregnancy.

Any woman who reported drinking at least 1.0 oz AA per day (≈2 standard drinks per day) or at least 2 instances of binge drinking (≥5 drinks per occasion) during the first trimester was invited to participate. Women initiating antenatal care who reported no binge drinking and minimal alcohol use (<0.5 oz AA/ day) were invited to participate as controls. In this sample all but one of the mothers in the control group reported abstaining from drinking during pregnancy; the single control mother who drank consumed only 2 drinks/occasion on 2–3 days during the pregnancy. Smoking was recorded as the number of cigarettes smoked per day. Participants were excluded if they were <18 years of age, presented with chronic medical problems including epilepsy, diabetes, HIV, or cardiac problems. Infant exclusionary criteria were major chromosomal anomalies, neural tube defects, multiple births, and seizures.

In 2005, we organized a clinic in which the children in the study were each independently examined by two expert dysmorphologists (E.H. Hoyme, M.D., and L.K. Robinson, M.D.) for growth and FAS related dysmorphic features using a standard protocol (Hoyme et al., 2005) based on the Revised Institute of Medicine criteria (Jacobson et al., 2008). Case conferences were held in which the dysmorphologists, SWJ, JLJ, and CDM determined which of the children met criteria for FAS or PFAS. Children who did not meet criteria for FAS of PFAS were designated as either non-syndromal heavily exposed (HE) or controls, depending on the maternal alcohol history. Five children who could not attend the 2005 clinic were examined by another expert FASD dysmorphologist (N. Khaole, M.D.), whose diagnoses were all subsequently confirmed by examinations conducted in follow-up clinics we held with the same dysmorphologists in 2009 and with HEH in 2013 and 2016.

### Magnetic resonance image (MRI) acquisition

Magnetic resonance image (MRI) scans were acquired at the Cape Universities Brain Imaging Centre (CUBIC) using a 3T Siemens Allegra MRI scanner (Siemens Medical Systems, Erlangen, Germany) for 81 right-handed children from this cohort at age 9–11 years. High-resolution T1-weighted images were obtained using a volumetric navigated (Tisdall et al., 2012) multi-echo magnetization prepared rapid gradient echo (MEMPRAGE) sequence optimized for morphometric neuroanatomical analysis using FreeSurfer software (van der Kouwe et al., 2008). Imaging parameters were: FOV 256 × 256 mm, 128 sagittal slices, TR 2,530 mm, TE 1.53/3.21/4.89/6.57 mm, TI 1,100 ms, flip angle 7°, voxel size 1.3 × 1.0 × 1.3 mm^3^, acquisition time 8:07 min. The volumetric navigator provides real-time motion tracking and correction to reduce artifacts resulting from motion.

Human subject participation was approved by the ethics committees of the University of Cape Town Faculty of Health Sciences and Wayne State University. Mothers provided written informed consent and children, oral assent. Examiners were blind with respect to PAE history and FASD diagnosis, except in the most severe cases of FAS. Total intracranial volumes (TIV) were obtained from FreeSurfer (Dale et al., 1999; Fischl et al., 2002). Called eTIV or ICV, within FreeSurfer, the programme calculates TIV by using an atlas scaling factor that it obtains from registering the images from the sample to an average template and using that scaling factor to estimate each participant's TIV (Buckner et al., 2004, see also http://surfer.nmr.mgh.harvard.edu/fswiki/eTIV).

### Manual tracing protocol

The CC and left and right caudate nuclei, nucleus accumbens and hippocampi were manually traced on the MR images using Multitracer (Woods, 2003) software on a tablet laptop (Lenovo ThinkPad X200, Wacom digitizer pen enabled). A single blinded investigator performed all manual tracings (SCB). Tracing was supervised and reviewed by CW, the senior neuroanatomist at the University of Cape Town. All structures were traced in the coronal plane, with the exception of the CC that was traced in the sagittal plane. Orthogonal planes were used to inform border delineation. “Frust” volumes were recorded for each structure, which assumes that a contour applies to the center of the slice on which it is drawn and that the square root of the cross-sectional area changes linearly across the slice thickness (Woods, 2003).

**Figure 1:**
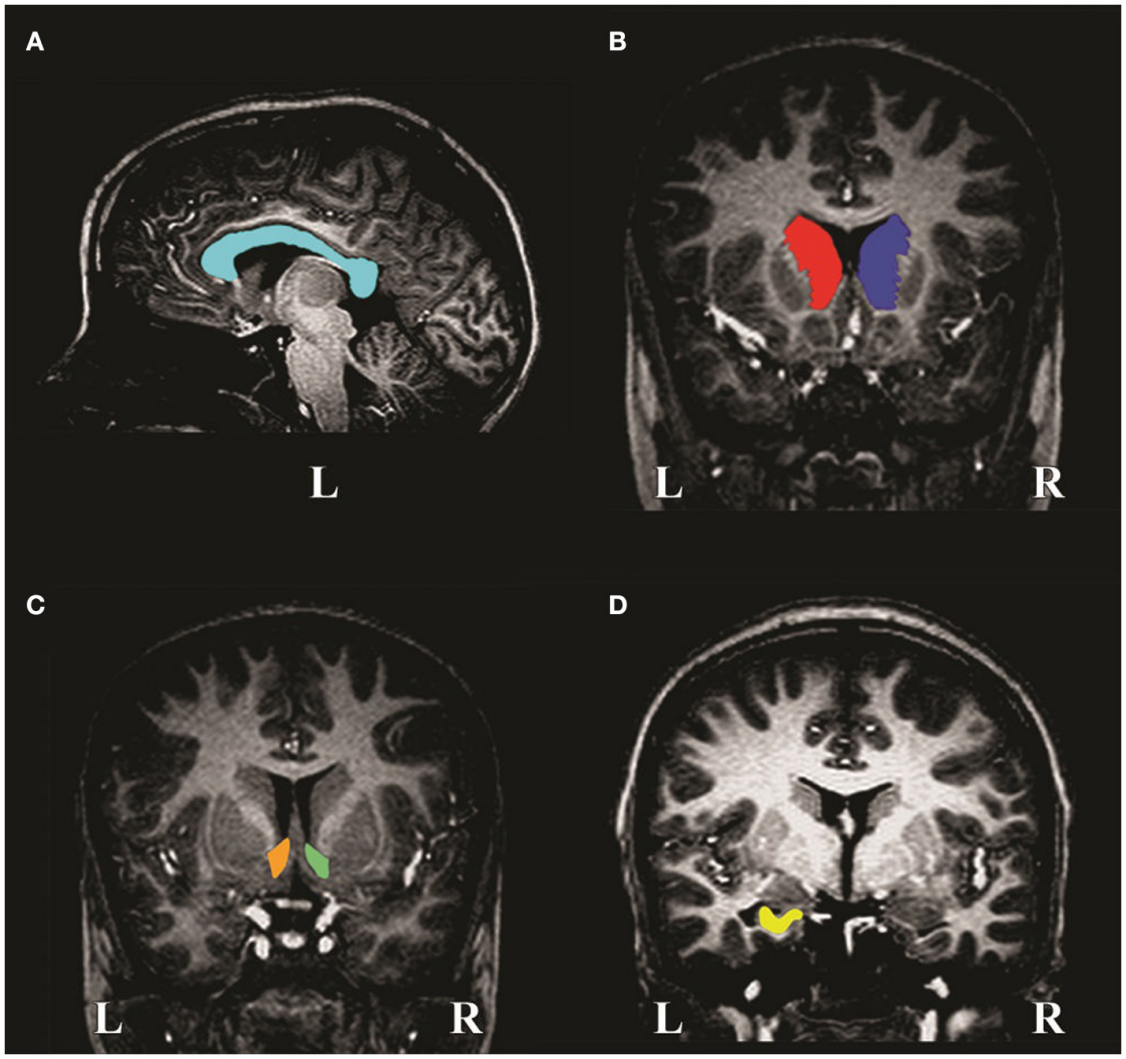
MR image slices of a non-exposed control child showing manual tracings of the **(A)** corpus callosum, **(B)** left and right caudate nuclei, **(C)** left and right nucleus accumbens and **(D)** left hippocampus. L and R denote left and right, respectively.

The CC (Figure 1A) is clearly visible as a curved WM structure near the midline slice. The superior border is the cingulate gyrus and the CSF (cerebrospinal fluid) of the lateral and posterior fissure form the inferior border. The midline slices (about slices 127–130) were determined by locating the smallest area of the thalamus. Bookstein et al. (2002) used the term “midline” to denote the slices located most medially in the CC (i.e., along the midline of the brain). The CC was traced on two contiguous midline slices. These were averaged and volume calculated.

The caudate nucleus (Figure 1B) is a gray matter (GM) structure that lies on the lateral wall of the lateral ventricle and is traced until it disappears. The medial border is the lateral ventricle and the lateral border is formed by WM. The inferior border is the nucleus accumbens (Figure 1C), which can only be differentiated histologically by cytoarchitecture, however, it can be removed from the caudate nucleus reliably by drawing a straight line from the inferior border of the lateral ventricle to the bottom-most border of the internal capsule. Laterally, it is separated from the putamen by a line descending from the internal capsule. It was traced until the anterior commissure bridges the midline.

The anterior border of the hippocampus (Figure 1D) is the inner surface of the alveus. Tracing proceeds past the crura of the fornices until hippocampal GM disappears. The medial border includes the subiculum to the supramedial border of WM in the temporal lobe. The lateral border is the inner surface of the alveus within the temporal horn of the lateral ventricle. Inferiorly, the boundary is the interface between the WM and GM.

Three participants (2 HE; 1 control) were excluded due to pathology (unrelated to the research question) and 7 (1 FAS; 2 HE; 4 control) due to compromised image quality. Pathology included one scan where there were hypointense inclusions on the superior surface of the right lateral ventricle (indicative of a potentially conflicting diagnosis), and two scans where the lateral ventricles were occluded, making delineation of caudate nuclei and nucleus accumbens problematic.

### Statistical analyses

Analysis of variance (ANOVA) was performed for each region of interest (ROI) to examine whether structural volumes differed between diagnostic groups. Pearson correlation was used to examine the relations of ROI volumes to each of the potential confounders. Potential control variables included TIV, child's sex and age (years) at scan, socioeconomic status (SES; Hollingshead, 2011), maternal smoking during pregnancy (cigarettes/day), and postnatal lead exposure (ug/dl) obtained from a blood sample at 5 years. ANCOVA was used to examine whether volumetric group differences remained significant after controlling for confounders even weakly (*p* < 0.10) related to the outcome. Although marijuana (days/week) and cocaine (days/week) were considered, only 5 mothers in this sample reported marijuana use and 1 cocaine. Any observed between-group differences were, therefore, rerun excluding children born to these mothers to see whether the effects persisted without them.

The association between continuous measures of PAE (both at conception and across pregnancy) and structural volumes were examined using Pearson correlation. Hierarchical multiple regression analyses were used to control for confounders. The alcohol measure was entered in the first step of each analysis for each outcome. Total intracranial volume (TIV) was then added to the regressions for the regional volumes to determine if the exposure affected the regional volume over and above its effect on total brain volume. Control variables related to the outcome at less than *p* < 0.10 were entered in the third step to determine if the effect of the prenatal alcohol measure on structure volume continued to be significant after statistical adjustment for these potential confounders. Observed associations with extent of alcohol exposure were rerun excluding children born to mothers who used marijuana or cocaine during pregnancy to see whether effects remained without them.

Pearson correlations were used to examine whether alcohol-related volumetric changes were associated with performance on the WISC-IV IQ and CVLT-C, two outcomes known to be related to PAE. Mediation of effects of PAE on these cognitive outcomes by structural volumes associated with PAE was examined using multiple regression. The effect of PAE on the cognitive outcome was entered in Step 1 of the regression; the structure volume, in Step 2. Mediation was inferred if the effect of PAE was significantly reduced when the structure volume was entered in Step 2, based on the Clogg test (Clogg et al., 1992). Table 1 shows a list of abbreviations used.

**Table 1:** List of abbreviations used.

PAE	Prenatal alcohol exposure
FAS	Fetal alcohol syndrome
PFAS	Partial fetal alcohol syndrome
HE	Heavily exposed non-syndromal participants
FAS/PFAS	The group comprising children with either a diagnosis of FAS or PFAS
AA	Absolute alcohol (oz)
TIV	Total intracranial volume
CC	Corpus callosum
Caudate	Caudate nucleus
NA	Nucleus accumbens

## Results

Following the exclusions described above, we report data for 71 children. Demographic characteristics are presented in Table 2. Although the FASD diagnostic groups did not differ by sex, prenatal exposure to cigarettes, marijuana, or cocaine, or childhood lead exposure, children in the HE group were slightly older than those in the FAS and PFAS groups. As expected, children in both the FAS and PFAS groups had lower IQs than the HE children; those in the PFAS group also had lower scores than the controls. Total brain volumes were smaller in the children with FAS compared to all other groups. SES was lower for mothers of children with FAS or PFAS than mothers of children from the HE and control groups.

**Table 2:** Sample characteristics (*N* = 71).

				**Exposed**								
		**Control**		**HE**		**PFAS**		**FAS**		**TOTAL**		***F* or *X^2^***
		**(*n* = 19)**		**(*n* = 24)**		**(*n* = 19)**		**(*n* = 9)**		**(*N* = 71)**		
**CHILD**												
Sex: Male	*n*(%)	9	(47%)	15	(63%)	11	(58%)	4	(44%)	39	(55%)	1.46
Age (yr) at scan^a^	Mean(SD)	10.6	(0.5)	11.0	(0.7)	10.5	(0.4)	10.4	(0.9)	10.7	(0.7)	2.84^*^
WISQ-IV IQ^b^	Mean(SD)	73	(10.7)	76	(11.3)	64	(10.2)	65	(13.1)	71	(12.1)	5.44^**^
Total intracranial volume (x10^6^ mm^3^)^c^	Mean (SD)	1.3	(0.1)	1.4	(0.2)	1.3	(0.1)	1.2	(0.1)	1.3	(0.1)	3.24^*^
**MATERNAL**												
Socioeconomic status^d^	Mean(SD)	23.5	(1.9)	24.0	(1.7)	15.2	(1.5)	16.9	(3.6)	20.6	(9.0)	5.44^**^
Cigarettes/day	Median(IQR)	0.0	(5.0)	5.3	(7.8)	7.5	(7.0)	9.0	(5.5)	5.0	(10.0)	1.34
Marijuana (yes)	*n*(%)	0	(0%)	2	(8%)	2	(11%)	1	(11%)	5	(7%)	2.08
Cocaine (yes)	*n*(%)	0	(0%)	0	(0%)	1	(5%)	0	(0%)	1	(1%)	2.78
Lead (ug/dl)	Mean(SD)	9.2	(3.5)	9.3	(3.3)	11.5	(6.5)	11.7	(4.3)	10.2	(4.6)	1.47
**ALCOHOL AT CONCEPTION**												
AA/day (oz)^e^	Median(IQR)	0.0	(0.0)	0.5	(1.0)	1.2	(1.2)	1.4	(1.6)	0.6	(1.3)	26.52^***^
AA/drinking day (oz)^f^	Mean(SD)	0.06	(0.3)	2.8	(2.2)	4.0	(2.1)	5.7	(2.8)	2.8	(2.7)	21.33^***^
Drinking days/wk^g^	Mean(SD)	0.0	(0.1)	1.3	(1.1)	2.6	(1.4)	2.8	(1.9)	1.5	(1.6)	18.88^***^
**ALCOHOL ACROSS PREGNANCY**												
AA/day (oz)^h^	Median(IQR)	0.0	(0.0)	0.2	(0.7)	1.0	(0.6)	0.8	(1.8)	0.3	(1.0)	22.73^***^
AA /drinking day (oz)^i^	Mean(SD)	0.06	(0.3)	3.4	(2.4)	3.8	(1.7)	4.9	(1.8)	2.8	(2.5)	21.83^***^
Drinking days/wk^j^	Mean(SD)	0.0	(0.0)	1.0	(0.8)	2.0	(1.0)	2.1	(1.8)	1.1	(1.2)	17.90^***^

*For skewed data medians and interquartile ranges (IQR) were used. HE, heavily exposed non-syndromal; FAS, fetal alcohol syndrome; PFAS, partial FAS; WISC-IV, Wechsler Intelligence Scales for Children-Fourth Edition*.

a *HE > FAS, PFAS (both p < 0.05)*.

b *FAS < HE (p = 0.011); PFAS < HE, Control (both p ≤ 0.01)*.

c *FAS < PFAS (p = 0.018), HE (p = 0.003), Control (p = 0.038)*.

d *Hollingshead, 2011; FAS < HE, Control (both p ≤ 0.05); PFAS < HE, Control (both p < 0.01)*.

e *Control < FAS, PFAS, HE (all p < 0.001); HE < FAS, PFAS (both p < 0.001)*.

f *Control < FAS, PFAS, HE (all p < 0.001); HE < FAS (p < 0.001); PFAS < FAS (p = 0.027)*.

g *Control < FAS, PFAS, HE (all p ≤ 0.001); HE < FAS, PFAS (both p < 0.01)*.

h *Control < FAS, PFAS, HE (all p < 0.001); HE < FAS, PFAS (both p ≤ 0.001)*.

i *Control < FAS, PFAS, HE (all p < 0.001); HE < FAS (p = 0.036)*.

j *Control < FAS, PFAS, HE (all p ≤ 0.001); HE < FAS, PFAS (both p < 0.01)*.

* *p ≤ 0.05*,

** *p ≤ 0.01*,

*** *p ≤ 0.001*.

As expected, all of the alcohol measures for the control group were statistically lower compared to the alcohol-using groups (Table 2). In addition, children with FAS were more exposed than HE children on all measures. They were also exposed to more alcohol/drinking day at conception than those in the PFAS group. At conception and across pregnancy, AA/day and drinking days/week for the PFAS group were also significantly higher than for the HE group. Although number of days of alcohol use was reduced from 1.5 (at conception) to 1.1 (across pregnancy) day/week on average [*t*_(68)_ = 3.88, *p* < 0.001), the number of drinks/occasion remained virtually unchanged, [*t*_(69)_ = −0.33, *p* = 0.741]. Moreover, alcohol consumption at conception was highly correlated with alcohol use throughout pregnancy (*r* = 0.923). Due to the small sample size of the FAS group, the two syndromal groups, FAS and PFAS, were combined for subsequent volumetric analyses.

Volumes of traced regions are reported for each diagnostic group in Table 3 and associations of control variables with structural volumes in Table 4. Children in the FAS/PFAS group had smaller right hippocampi compared to both the HE and control children, but this effect was no longer significant after adjustment for TIV. Volumes of four regions were smaller in girls than in boys. Among the remaining potential confounders, smoking during pregnancy and postnatal lead exposure were related to size in one region—the CC.

**Table 3:** Comparison of structure volumes by diagnostic group.

**ROI**	**Control (*n* = 1)**		**HE (*n* = 24)**		**FAS/PFAS (*n* = 28)**		**Total (*N* = 71)**		***p***	***p*^1^**	***p*^2^**
**GRAY MATTER ROIs**											
L Caudate^a^	3,929	(563)	3,798	(543)	3,581	(563)	3,748	(567)	0.10	0.11	0.11
R Caudate^a^	3,936	(582)	3,804	(552)	3,662	(607)	3,783	(584)	0.29	0.26	0.24
L NA^a^	542	(117)	549	(72)	503	(89)	529	(93)	0.16	0.37	0.28
R NA^a^	490	(95)	490	(83)	467	(90)	481	(88)	0.57	0.78	0.44
L Hippocampus	2,400	(224)	2,392	(346)	2,277	(360)	2,349	(325)	0.33	0.53	0.53
R Hippocampus	2,432	(185)	2,469	(302)	2,266	(318)	2,379	(294)	**0.03^#^**	0.09	0.09
**WHITE MATTER ROI**											
CC^b^^,^ ^c^	564	(64)	570	(107)	525	(79)	551	(88)	0.13	0.21	0.52

*Values are mean(SD). Volumes are in mm^3^. NA, nucleus accumbens; CC, corpus callosum; HE, heavily exposed non-syndromal; FAS, fetal alcohol syndrome; PFAS partial (FAS). Bold print denotes significance at p ≤ 0.05*.

# FAS/PFAS < HE non-syndromal (p = 0.012) and Control (p = 0.052). p^1^ after controlling for TIV. p^2^ after controlling for TIV, as well as (

a ) sex of child, (

b ) cigarettes/day during pregnancy, (

c *) lead concentration (ug/dl)*.

**Table 4:** Correlations of control variables with structure volumes.

**ROI (mm^3^)**	**TIV**	**Sex**	**Cigarettes/day during pregnancy**	**Socioeconomic status**	**Lead concentration (ug/dl)**	**Age (year)**
**GRAY MATTER ROIs**						
L Caudate	**0.520**	**−0.215**	−0.113	0.087	−0.168	−0.082
	**(0.001)**	**(0.079)**	(0.350)	(0.468)	(0.161)	(0.499)
R Caudate	**0.496**	**−0.222**	−0.066	0.057	−0.126	−0.069
	**(0.001)**	**(0.052)**	(0.584)	(0.638)	(0.296)	(0.565)
L NA	**0.421**	**−0.367**	0.004	0.061	0.120	−0.080
	**(0.001)**	**(0.002)**	(0.975)	(0.614)	(0.321)	(0.505)
R NA	**0.504**	**−0.381**	0.041	−0.012	0.148	−0.081
	**(0.001)**	**(0.001)**	(0.735)	(0.920)	(0.218)	(0.502)
L Hippocampus	**0.349**	−0.137	−0.109	−0.068	−0.141	0.058
	**(0.003)**	(0.255)	(0.364)	(0.574)	(0.242)	(0.630)
R Hippocampus	**0.402**	−0.167	−0.076	−0.059	−0.043	0.016
	**(0.002)**	(0.307)	(0.526)	(0.625)	(0.719)	(0.892)
**WHITE MATTER ROI**						
CC	0.184	0.043	**–0.325**	0.166	**−0.209**	0.087
	(0.124)	(0.723)	**(0.006)**	(0.166)	**(0.080)**	(0.470)

*Bold print denotes significance at p ≤ 0.1. Values are Pearson r (p). NA, nucleus accumbens; CC, corpus callosum. Sex (male = 1, female = 2)*.

Table 5 presents the results of the multiple regression analyses examining the relation of continuous measures of PAE to the structural volumes. PAE was associated with volume reductions bilaterally in the caudate and hippocampus and smaller CC, but the effect on the CC was no longer significant after controlling for smoking during pregnancy and postnatal lead exposure. When the regression for CC was rerun controlling only for lead, the effect of absolute alcohol/day remained significant (β = −0.281, *p* = 0.022). In a regression of the effect of alcohol and smoking on CC volume, the effects of both of these exposures fell just short of significance (alcohol β = −0.236, *p* = 0.065; smoking β = −0.217, *p* = 0.089), suggesting that neither effect was attributable to the effect of the other exposure.

**Table 5:** Association of alcohol consumption measures with structure volumes.

**ROI**	**Absolute alcohol/day**			**Drinking days/week**		
	***r* (*p*)**	***β*_1_ (*p*)**	***β*_2_ (*p*)**	***r* (*p*)**	***β*_1_ (*p*)**	***β*_2_ (*p*)**
**GRAY MATTER ROIs**						
L Caudate^a^	**−0.366**	**−0.291**	**−0.366**	**−0.399**	**−0.317**	**−0.400**
	**(0.002)**	**(0.005)**	**(0.001)**	**(0.001)**	**(0.002)**	**(<0.001)**
R Caudate^a^	**−0.311**	**−0.241**	**−0.311**	**−0.333**	**−0.256**	**−0.334**
	**(0.008)**	**(0.023)**	**(0.007)**	**(0.005)**	**(0.016)**	**(0.004)**
L NA^a^	−0.142	−0.090	−0.142	−0.173	−0.117	−0.175
	(0.239)	(0.404)	(0.204)	(0.149)	(0.279)	(0.117)
R NA^a^	−0.085	−0.016	−0.085	−0.115	−0.039	−0.116
	(0.481)	(0.878)	(0.446)	(0.341)	(0.711)	(0.298)
L Hippocampus	**−0.295**	**−0.245**	**−0.245**	**−0.313**	**−0.257**	**−0.257**
	**(0.012)**	**(0.031)**	**(0.031)**	**(0.008)**	**(0.024)**	**(0.024)**
R Hippocampus	**−0.275**	**−0.215**	**−0.215**	**−0.281**	−0.214	−0.214
	**(0.020)**	**(0.054)**	**(0.054)**	**(0.018)**	(0.056)	(0.056)
**WHITE MATTER ROI**						
CC^b^^,^ ^c^	**−0.335**	**−0.313**	−0.179	**−0.353**	**−0.330**	−0.206
	**(0.004)**	**(0.008)**	(0.176)	**(0.003)**	**(0.005)**	(0.116)

*Bold print denotes significance at p ≤ 0.05*.

*β_1_ is the standard regression coefficient adjusted for TIV*.

β_2_ is the standard regression coefficient controlling for TIV, as well as (

a ) sex of child, (

b ) cigarettes/day during pregnancy, (

c *) lead concentration (ug/dl)*.

Prenatal alcohol exposure (PAE) was associated with lower IQ (*r* = −0.402; *p* = 0.001) and CVLT-C (*r* = −0.248; *p* = 0.045) scores. Larger CC was associated with higher IQ for the sample as a whole and with better CVLT-C performance for the FAS/PFAS group (Table 6). Unexpectedly, smaller hippocampi were associated with better CVLT-C scores for the FAS/PFAS group.

**Table 6:** Associations of structure volumes with IQ and CVLT-C scores.

**ROI**	**IQ**				**CVLT-C**			
	**Control**	**HE**	**FAS/PFAS**	**Total**	**Control**	**HE**	**FAS/PFAS**	**Total**
	***n*** = **19**	***n*** = **24**	***n*** = **28**	***N*** = **71**	***n*** = **19**	***n*** = **22**	***n*** = **25**	***N*** = **66**
L Caudate	−0.064	−0.190	0.164	0.081	0.112	−0.359	0.045	−0.013
	(0.795)	(0.375)	(0.405)	(0.504)	(0.647)	(0.101)	(0.832)	(0.916)
R Caudate	−0.118	−0.118	0.128	0.050	0.065	−0.232	0.098	−0.021
	(0.629)	(0.584)	(0.516)	(0.682)	(0.791)	(0.299)	(0.641)	(0.870)
L Hippocampus	0.136	−0.069	0.032	0.087	0.089	−0.286	**−0.439**	−0.233
	(0.580)	(0.749)	(0.871)	(0.469)	(0.716)	(0.196)	**(0.028)**	(0.060)
R Hippocampus	−0.064	−0.088	−0.125	0.055	0.251	−0.205	**−0.472**	−0.172
	(0.794)	(0.683)	(0.527)	(0.646)	(0.300)	(0.361)	**(0.017)**	(0.168)
CC	−0.122	0.324	**0.381**	**0.327**	−0.060	0.001	**0.389**	−0.060
	(0.619)	(0.122)	**(0.045)**	**(0.005)**	(0.808)	(0.997)	**(0.049)**	(0.808)

*NA, nucleus accumbens; CC, corpus callosum; HE, heavily exposed non-syndromal; FAS, fetal alcohol syndrome; PFAS, partial (FAS). Bold print denotes significance at p ≤ 0.05. Values are Pearson r (p)*.

**Figure 2:**
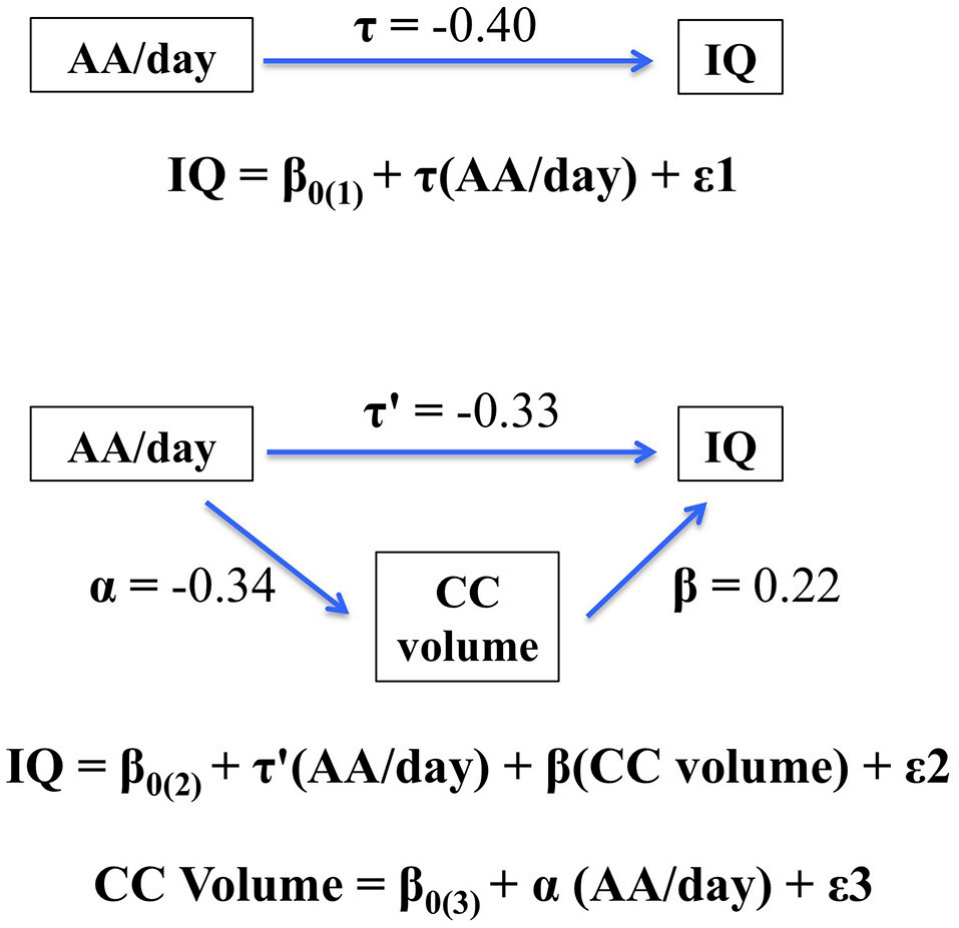
Path model showing partial mediation of the association between AA/day and IQ by corpus callosum (CC) volume. The figure shows that the effect of prenatal alcohol exposure on IQ is partially mediated by the fetal alcohol-related corpus callosum volume reduction. When CC size was added in Step 2 of the regression analysis, the effect of AA/day on IQ was reduced from −0.40 to −0.33, a reduction that was statistically significant, Clogg (*t* = −2.86, *p* < 0.01).

The hypothesis that CC size would mediate the effect of PAE on IQ was tested using multiple regression. PAE was entered in Step 1. When CC size was added in Step 2 of the regression analysis, the effect of AA/day on IQ was reduced from −0.40 to −0.33, a reduction that was statistically significant, Clogg (*t* = −2.86, *p* < 0.01). These data thus indicate that the effect of PAE on IQ was partially mediated by the alcohol-related reduction in CC size (Figure 2).

## Discussion

This study shows that increasing PAE, both in terms of quantity and frequency, is associated with disproportionate volume reductions bilaterally in the caudate nuclei and hippocampi and smaller CC. By contrast, volume differences between FASD diagnostic groups were evident only in the right hippocampus. Smaller CC was associated with poorer IQ. Within the FAS/PFAS group, smaller CC was also associated with lower CVLT-C scores, but hippocampal volume reductions were associated with better CVLT-C performance. The results from our mediation analysis suggest that the adverse effect of PAE on IQ is partially attributable to the reductions in CC size.

When the regional volumetric measures were compared across the three diagnostic groups, no significant differences were seen after adjustment for TIV. By contrast, the continuous measures of PAE were associated with volumetric differences in all of the regions examined after adjustment for brain size, except the bilateral NA. These findings are consistent with other neuroimaging studies performed with children from the same Cape Town cohort, which found that continuous measures of PAE were more sensitive than diagnosis to fetal alcohol-related alterations in brain structure and function (De Guio et al., 2014; Meintjes et al., 2014; du Plessis et al., 2015).

Our finding of alcohol-related volumetric reductions bilaterally in the caudate nuclei are consistent with studies reporting decreased basal ganglia volume in FAS (Mattson et al., 1994, 1996, 1999, 2001; Archibald et al., 2001; Roussotte et al., 2012). Animal studies suggest that the physiological mechanisms underlying smaller striatal volumes may be explained by a reduction in dendritic tree formation rather than a reduction in number of parvalbumin interneurons (De Giorgio et al., 2012). The caudate nucleus is involved in many of the neurobehavioural deficits exhibited by children prenatally exposed to alcohol, including executive function domains, such as cognitive flexibility, concept formation and reasoning, planning and response inhibition (Mattson et al., 1999, 2001). This region has also been shown to be activated less by children with FASD during behavioral inhibition tasks (Fryer et al., 2007) and to mediate cognitive control and verbal learning in children with PAE (Fryer et al., 2012). Although the latter study reported that larger caudate nuclei were associated with better performance in verbal learning in alcohol-exposed children, we did not confirm that finding in our study.

The caudate nuclei may be more susceptible to the effects of PAE than the NA. It has been suggested that some midline brain structures are more affected by alcohol exposure during embryological development than others (Sulik et al., 1981; Zhou et al., 2003; Meintjes et al., 2014). Our results are consistent with those of Archibald et al. (2001), who also failed to find alcohol-related volume reductions in the NA using a similar tracing methodology. Because the NA can only truly be differentiated from the caudate nucleus histologically, the lack of association in both these studies may be due to poor sensitivity by these tracing methods to differentiate the NA from the caudate nuclei. Thus, although our results suggest that the NA may be relatively spared in PAE, further investigation using more sensitive methods, such as diffusion tensor imaging or higher resolution structural imaging, is needed.

Prenatal alcohol exposure (PAE) was also associated with volumetric reductions in both the left and right hippocampi–effects that remained significant after control for TIV. Reductions in pyramidal and granule cell number and density were observed in rats exposed prenatally to alcohol (Livy et al., 2003), which suggests that this may be the case in humans. The hippocampal formation has been shown to be particularly sensitive to PAE, showing a diverse range of negative effects and is one of the few areas of the brain which continues to exhibit these deficits as it develops into adulthood (see review, Gil-Mohapel et al., 2010). Associations of smaller hippocampi with better memory performance in typically-developing young adults (Van Petten et al., 2004) suggest a neural pruning process with age that may alter the relation of hippocampal volume in healthy controls as they grow older. Although our findings for the caudate nuclei, NA and CC are consistent with those of Archibald et al. (2001), who used a similar tracing protocol to ours, fetal alcohol-related differences in hippocampal volumes in their study did not survive after controlling for TIV. This disparity in results may be due to the wide range of ages included in that study (7–24 years) compared to the more limited range in ours (9–11 years). The observed association of smaller hippocampal volume with better learning and memory performance in the FAS/PFAS group in the current study was not expected at this age and is difficult to interpret.

Prenatal alcohol exposure (PAE) was associated with smaller CC after adjustment for TIV and postnatal lead exposure. However, regression analyses showed that the effects of smoking and alcohol on CC volume were confounded in this sample, and both fell slightly short of conventional levels of statistical significance when included in the same analysis. The effects of maternal smoking on the child's brain are not well understood, but decreases in CC size and changes in microstructure of other frontal WM structures have been linked with maternal smoking during pregnancy (Jacobsen et al., 2007; Jacobson et al., 2008; Paus et al., 2008). Given that the PAE and maternal smoking effects were similar in magnitude when adjusted for each other in the regression analysis, neither effect appears to be attributable to the effect of the other exposure, and a larger sample would likely have shown their effects to be independent.

The hypothesis that volumetric reductions in the caudate nuclei and hippocampi would mediate effects of PAE on cognitive performance was not supported. Volumetric reductions in the caudate nuclei did not relate to the cognitive outcomes; and, contrary to expectation, the hippocampal volumes in the FAS/PFAS group were inversely related to learning and memory performance.

In this study, larger CC area was associated with higher IQ scores and, amongst children in the FAS/PFAS group, with better learning and memory performance. Mediation analysis indicated that the effect of PAE on IQ was partially mediated by reductions in CC size. Our findings are consistent with several studies that have suggested that WM is more susceptible to the teratogenic effects of PAE than cortical GM (Archibald et al., 2001; Lebel et al., 2008; Spottiswoode et al., 2011). It is of interest that microstructural changes in two regions of the CC have also been found to mediate effects of PAE on IQ (Fan et al., 2016).

Morphology of the CC has been shown to have functional effects, with a thinner midline being associated with poorer motor activity and thicker than average midline being associated with poorer executive function (Bookstein et al., 2002). The CC is particularly important for functional outcomes requiring interhemispheric transfer of information, and it has been shown that even moderate exposure to alcohol may influence this process (Roebuck et al., 2002; Dodge et al., 2009). Since cognitive function assessed on IQ tests depends on a broad range of cognitive domains (Wechsler, 2003; Willoughby et al., 2008) utilizing a range of GM areas that communicate via axons in the CC, poorer interhemispheric transfer could contribute to lower IQ (Dodge et al., 2009). Smaller CC size may be due to either poorer myelination or fewer axons in the CC, resulting in slower signal processing or communication with greater numbers of GM cell bodies, respectively. CC volume reductions may also be due to changes to CC projection neurons, as reductions in size, number and location of these cells were observed in rats prenatally exposed to alcohol (Livy and Elberger, 2008). However, as no one animal model exhibits all the features of FASD (Cudd, 2005), further investigation into the underlying physiological mechanisms in humans is needed to better understand the links between CC and behavioral outcomes. As such, it is not surprising that the most heavily exposed children, those in the FAS/PFAS group, had the smallest CC areas and the lowest IQ scores.

One potential limitation of this study that is common to other longitudinal PAE studies is that it relies on the mother's report to assess alcohol consumption. However, we have previously validated the TLFB interview used in this study in relation to levels of fatty acid ethyl ester metabolites in meconium samples obtained from newborns in this cohort (Bearer et al., 2003). In addition, this TLFB interview has been shown to predict a broad range of behavioral (Jacobson et al., 2002, 2008; Lewis et al., 2015; Lindinger et al., 2016; Fortin et al., 2017) and neuroimaging outcomes (De Guio et al., 2014; Meintjes et al., 2014; du Plessis et al., 2015; Jacobson et al., 2017). A second limitation relates to the difficulty assessing effects of timing of exposure in a human study. Animal models have demonstrated that timing of exposure is a major determinant of specific longterm neurobehavioral outcomes, since different brain regions are susceptible to PAE during unique sensitive periods in their development (Livy et al., 2003; Valenzuela et al., 2012; Sadrian et al., 2014). Unfortunately, multicollinearity between maternal alcohol use at conception and later in pregnancy in this cohort, as in most human samples, was too high to discriminate effects attributable to timing of exposure. Polysubstance abuse is another common limitation in PAE studies. The current study assessed potential confounding by prenatal cocaine, marijuana and smoking exposure in order to address this problem; none of these exposures were related to the outcomes examined. Strengths of this study include the use of a sample size that was large relative to other manual tracing studies, limited age range, a population that is geographically and culturally homogenous albeit diverse in terms of genetic ancestry, and the availability of continuous measures of prospectively collected alcohol consumption.

In conclusion, this study, which used manual tracing, confirms previous reports linking PAE to decreases in subcortical volumes in the caudate nucleus and hippocampus after adjustment for TIV and also showed that PAE is associated with a disproportionate reduction in CC size. This is the first study to provide evidence that fetal alcohol-related reductions in CC partially mediate effects of PAE on IQ. This finding adds to the growing body of evidence suggesting that WM may be particularly vulnerable to the teratogenic effects of PAE on development of the fetal brain.

## Ethics statement

Ethical approval for the study was acquired from the University of Cape Town Faculty of Health Sciences Human Research Ethics Committee and the Wayne State University Institutional Review Board. Informed written consent was obtained from the mothers and oral assent from the children in accordance with the Declaration of Helsinki.

## Author contributions

SB performed the tracing of the volumes under the supervision of CW and with the assistance of SR. She also reviewed the literature, performed the data analyses, contributed to the interpretation and wrote up the findings. EM provided overall project supervision, collaborated on the design of the neuroimaging study, the data analysis, interpretation and write-up of the findings. SJ and JJ designed the study, supervised recruitment and maternal interviews and child assessments, collaborated on the data analysis, the interpretation of the findings and write-up of the paper. CM administered the maternal interviews, which included sociodemographic information and alcohol, smoking and drug ascertainment. NL and CL performed neuropsychological assessments.

### Conflict of interest statement

The authors declare that the research was conducted in the absence of any commercial or financial relationships that could be construed as a potential conflict of interest.
